# Focused ultrasound-mediated drug delivery to pancreatic cancer in a mouse model

**DOI:** 10.1186/2050-5736-1-11

**Published:** 2013-07-01

**Authors:** Natalya Rapoport, Allison Payne, Christopher Dillon, Jill Shea, Courtney Scaife, Roohi Gupta

**Affiliations:** 1 Department of Bioengineering, University of Utah, 36 S. Wasatch Dr., Room 3100, Salt Lake City, UT 84112, USA; 2 Department of Radiology, University of Utah, Salt Lake City, UT 84112, USA; 3 Department of Surgery, University of Utah, Salt Lake City, UT 84112, USA; 4 Current address: Department of Radiation Oncology, Fox Chase Cancer Center, P0103, 333 Cottman Avenue, Philadelphia, PA 19111, USA

**Keywords:** Ultrasound-mediated drug delivery, Perfluorocarbon nanoemulsions, MRgFUS, Pancreatic cancer

## Abstract

**Background:**

Many aspects of the mechanisms involved in ultrasound-mediated therapy remain obscure. In particular, the relative roles of drug and ultrasound, the effect of the time of ultrasound application, and the effect of tissue heating are not yet clear. The current study was undertaken with the goal to clarify these aspects of the ultrasound-mediated drug delivery mechanism.

**Methods:**

Focused ultrasound-mediated drug delivery was performed under magnetic resonance imaging guidance (MRgFUS) in a pancreatic ductal adenocarcinoma (PDA) model grown subcutaneously in nu/nu mice. Paclitaxel (PTX) was used as a chemotherapeutic agent because it manifests high potency in the treatment of gemcitabine-resistant PDA. Poly(ethylene oxide)-co-poly(d,l-lactide) block copolymer stabilized perfluoro-15-crown-5-ether nanoemulsions were used as drug carriers. MRgFUS was applied at sub-ablative pressure levels in both continuous wave and pulsed modes, and only a fraction of the tumor was treated.

**Results:**

Positive treatment effects and even complete tumor resolution were achieved by treating the tumor with MRgFUS after injection of nanodroplet encapsulated drug. The MRgFUS treatment enhanced the action of the drug presumably through enhanced tumor perfusion and blood vessel and cell membrane permeability that increased the drug supply to tumor cells. The effect of the pulsed MRgFUS treatment with PTX-loaded nanodroplets was clearly smaller than that of continuous wave MRgFUS treatment, supposedly due to significantly lower temperature increase as measured with MR thermometry and decreased extravasation. The time of the MRgFUS application after drug injection also proved to be an important factor with the best results observed when ultrasound was applied at least 6 h after the injection of drug-loaded nanodroplets. Some collateral damage was observed with particular ultrasound protocols supposedly associated with enhanced inflammation.

**Conclusion:**

This presented data suggest that there exists an optimal range of ultrasound application parameters and drug injection time. Decreased tumor growth, or complete resolution, was achieved with continuous wave ultrasound pressures below or equal to 3.1 MPa and drug injection times of at least 6 h prior to treatment. Increased acoustic pressure or ultrasound application before or shortly after drug injection gave increased tumor growth when compared to other protocols.

## Introduction

Chemotherapy remains the treatment of choice for many types of cancer. During the last decade, progress in nanotechnology has enabled tumor-targeted delivery of anticancer drugs, which simultaneously decreased side effects and increased drug concentration in tumor tissue. However, the dream of a ‘magic bullet’ that would exclusively target the tumor remains elusive. In pursuit of this goal, a number of groups have been directing their efforts to increase the degree of drug tumor-targeting using ultrasound-mediated drug delivery. In this approach, drug delivery with nanoparticles is combined with tumor-directed ultrasound that affects both the drug carrier and tumor tissue. The use of ultrasound triggers drug release from the nanoparticle carrier and increases drug and carrier extravasation and deposition in tumor cells.

Focused ultrasound (FUS) has a number of advantages when compared with other physical methods used in tumor therapy: it can penetrate deep into the body, can be focused in a region with a diameter of approximately 1 mm, can be carefully controlled, and is completely non-invasive. FUS has both thermal (local heating of tissue to hyperthermia or ablative temperatures) and non-thermal mechanisms (i.e., cavitation and radiation force). These mechanisms are expected to work synergistically to trigger drug release from the carrier, increase extravasation and internalization of carrier and drug, and increase drug diffusion in tumor tissue, which ultimately results in an enhanced treatment outcome.

Development of real-time imaging methods such as magnetic resonance imaging (MRI) or ultrasound imaging allows precise spatiotemporal control of FUS-mediated drug delivery. Imaging assists in the identification of the target, guidance of ultrasound action, and evaluation of therapeutic efficacy. Moreover, real-time temperature measurements during treatment using MRI thermometry provide data that can be used in a feedback controller to enable greater control of the energy delivery [1,2].

Bioeffects of ultrasound in drug delivery may be enhanced by the application of microbubbles, which have been widely used as ultrasound contrast agents. This novel application of microbubbles is associated with enhanced cavitation [3-13]; microbubbles have been successfully used for opening blood/brain and blood/retinal barriers [7,9,14,15] or enhancing thrombus dissolution [16-18]. However, the size of microbubbles (usually 2 to 10 microns) precludes their extravasation into the tumor tissue.

We have developed drug-loaded perfluorocarbon nanoemulsions that serve as nanoscale microbubble precursors [19-24]. Due to their size (200 to 300 nm, i.e., at least the order of magnitude is smaller than microbubbles), nanodroplets can extravasate into the tumor tissue. Under the action of tumor-directed ultrasound, perfluorocarbon droplets convert into microbubbles [25-28]. Droplet-to-bubble conversion results in a release of an encapsulated drug [21,23,29-33]. Using paclitaxel-loaded perfluorocarbon nanoemulsions, successful therapy of breast, ovarian, and pancreatic cancer has been achieved in preclinical studies using animal models; in these studies, unfocused ultrasound was blindly applied to the tumor region [21,22].

Despite these successes, many aspects of the mechanisms involved in ultrasound-mediated therapy remain obscure. In particular, the relative roles of drug and ultrasound applications, the effect of the time of ultrasound application, and the effect of tissue heating are not yet clear. The current study was undertaken with the goal to clarify these aspects of the FUS-mediated drug delivery mechanism. A pancreatic ductal adenocarcinoma (PDA) model grown subcutaneously in nu/nu mice was used as a tumor model.

PDA is the fourth leading cause of death from cancer in the USA, and novel approaches to PDA therapy are urgently needed. Poor PDA response to conventional chemotherapy is at least partly accounted for by poor tumor vascularization and high content of stroma that precludes effective drug delivery and diffusion throughout tumor volume [34-44]. Since FUS-mediated drug delivery enhances drug extravasation and diffusion, this treatment modality is expected to have significant impact on the development of effective PDA therapy.

In this study, FUS-mediated drug delivery was performed under MRI guidance. Paclitaxel (PTX) was used as a chemotherapeutic agent because it manifested high potency in the treatment of gemcitabine-resistant PDA [45]. Poly(ethylene oxide)-co-poly(d,l-lactide) (PEG-PDLA) block copolymer stabilized perfluoro-15-crown-5-ether (PFCE) nanoemulsions or their mixtures with polymeric micelles were used as drug carriers [21,31,33]. As manifested by the appearance of harmonic frequencies in the fast Fourier transform emission spectra, PFCE nanodroplets were converted into microbubbles under ultrasound irradiation and underwent stable cavitation in both liquid emulsions and gel matrices [32]. Droplet-to-bubble conversion proceeded presumably via the evolution of dissolved oxygen into a separate phase [21].

The objective of this study is to better understand the advantages and limitations of the FUS-mediated drug delivery application to PDA therapy.

## Materials and methods

### Drug

PTX was used as a chemotherapeutic agent because it has manifested high potency in the treatment of gemcitabine-resistant PDA [45]. PTX was obtained from LC Laboratories (Woburn, MA, USA).

### Preparation of paclitaxel-loaded PFCE nanodroplets

**Figure 1:**
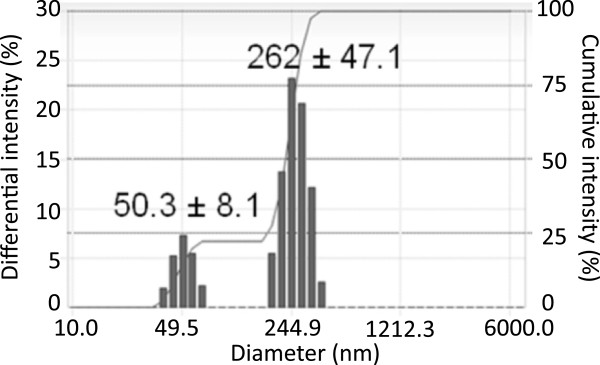
**A nanoparticle size distribution for 5% PEG-PDLA/1% PFCE formulation.** Fifty-nanometer particles are residual micelles; two-hundred sixty-two-nanometer particles are nanodroplets. Nanodroplet size may be decreased by increasing sonication pressure during emulsification. Micelle fraction can be decreased by decreasing copolymer concentration and/or increasing PFCE concentration [46]. PTX loading slightly increases nanodroplet sizes (e.g., from 260 to 280 nm).

PTX-loaded perfluorocarbon nanodroplets were manufactured from PTX-loaded micelles formed by the water-soluble, biodegradable block copolymer PEG-PDLA with a molecular weight of either block of 2,000 Da (Akina, Inc., West Lafayette, IN, USA). PTX-containing PEG-PLA micellar solutions were prepared by a solid dispersion technique [20]. Typically, 20 or 50 mg PEG-PDLA and 5 mg PTX were co-dissolved in 1 ml tetrahydrofuran (THF). The THF was then evaporated under gentle nitrogen stream at 60°C or pumped out at room temperature. PTX-loaded micelles were reconstituted by dissolving residual gel matrix in 1 ml phosphate buffered saline (PBS; pH 7.4). Then 10 μl PFCE (MW 580.01, Oakwoods Products, Inc., West Columbia, SC, USA) was introduced into micellar solution and emulsified by sonication on ice (VCX500, Sonics and Materials, Inc., CT, USA) to obtain paclitaxel-loaded droplets of the composition 2% or 5% PEG-PDLA/0.5% PTX/1% PFCE. The components of micellar or nanodroplet formulations were obtained from commercial sources and used without further purification. Micellar solutions and perfluorocarbon compounds were sterilized by filtration and mixed in a sterile test tube before being sonicated on ice for the generation of the nanoemulsion. The size of PFCE nanodroplets (both empty and drug loaded) was in the range 250 to 300 nm (Figure 1).

### Subcutaneous PDA MiaPaCa-2 tumor model

Human pancreatic cancer MiaPaCa-2 cells were obtained from the American Type Culture Collection (Rockville, MD, USA) and transfected with red fluorescence protein (RFP) [47]. Because only live cells generate RFP and therefore are fluorescent, the intravital whole mouse fluorescence imaging allowed the monitoring of tumor size and death of clusters of tumor cells. The excitation and emission peaks for the RFP were 563 and 587 nm, respectively.

Cells were maintained in Dulbecco's modified Eagle medium (DMEM) supplemented with 10% heat-inactivated fetal bovine serum (Gibco, Grand Island, NY, USA) at 37°C in a 5% CO_2_ incubator. Male nude mice between 6 to 8 weeks of age were utilized (NCr-Nu/Nu, National Cancer Institute, Frederick, MD, USA). For the tumor induction, mice were anesthetized with isoflurane and received a single subcutaneous injection of 1.5 × 10^6^ MiaPaCa-2 cells suspended in 150 μL of serum-free DMEM. Tumors were grown in either the shoulder or thigh region and allowed to progress until reaching an initial size of at least 175 mm^3^, at which point mice were randomly assigned to a treatment group. Since the tumor size of untreated animals roughly doubles every week, the target initial tumor size of 175 mm^3^ was often exceeded, and in the majority of animals, the initial tumor size was 200 to 300 mm^3^ immediately before treatment. To assess the effect of the initial tumor size on treatment outcome, tumors were allowed to grow to the volume of roughly 1,000 mm^3^ in a small subset of animals. All experiments were approved by the University of Utah Institutional Animal Care and Use Committee.

### MRgFUS treatments

At an assigned time point (2 or 8 h) before magnetic resonance-guided FUS (MRgFUS) therapy, mice were systemically injected through the tail vein with either empty (i.e., non-PTX-loaded) or PTX-loaded PFCE nanodroplets (PTX dose 40 mg/kg). In one experiment, the MRgFUS treatment was performed 10 min before drug injection.

The MRgFUS treatments were executed with a small animal MRgFUS system (Image Guided Therapy, Inc., Pessac, France) with a 16-element annular transducer (*f* = 3 MHz, *r*_c_ = 3.5 cm, FWHM = 1 × 3 mm) that could be translated in plane with piezo-ceramic motors. The system was placed in a Siemens 3 T Trio scanner, and temperature imaging was obtained using a 2D-segmented echo-planar imaging (EPI) sequence (TR/TE = 60/10 ms, FA = 15°, EPI = 3, fat saturation, 752 Hz/pixel, 1.4 s acquisition, 2 × 2 × 3 mm^3^ resolution, single slice). Temperatures were reconstructed using a referenceless algorithm [48] and were post-processed with zero-filled interpolation to yield 1 × 1 × 3 mm^3^ voxel spacing [49].

Four different acoustic peak pressure levels were applied: 2.4, 3.4, 4.2, and 4.8 MPa. These levels were calculated in water at the site of sonication assuming that the beam intensity is distributed evenly over the focal spot. Most experiments in the PTX-nanodroplet + MRgFUS group were performed with continuous wave (CW) ultrasound (*N* = 24); in parallel with CW experiments, some experiments were performed with pulsed ultrasound (*N* = 4), with pressure levels matching those of CW counterparts.

**Figure 2:**
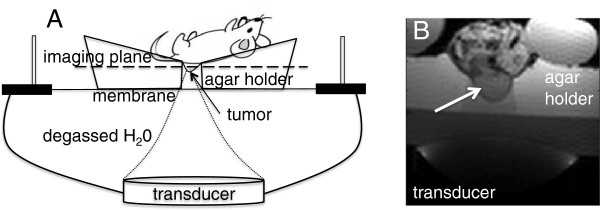
**Schematic representation and axial image of mouse on small-animal MRgFUS device. (A)** Schematic representation of the mouse positioning on the small animal MRgFUS device; **(B)** An axial image of mouse 59 on the small animal MRgFUS device with labeled transducer and agar holder. The white arrow indicates the tumor (initial size 455 mm^3^).

Treatments were conducted using the following protocol: the free-breathing anesthetized mouse (ketamine100 mg/kg, xylazine 20 mg/kg) was placed on the agar holder such that the tumor protruded through the hole as shown schematically in Figure 2A. In order to ensure an adequate acoustic window, the animal was always positioned with the tumor directly above the ultrasound transducer. An axial image of a mouse on the MRgFUS device placed in the magnet is shown in Figure 2B. The transducer and agar holder are shown. The mouse is flanked by warm-water-filled tubes to help regulate its body temperature during the treatment.

**Figure 3:**
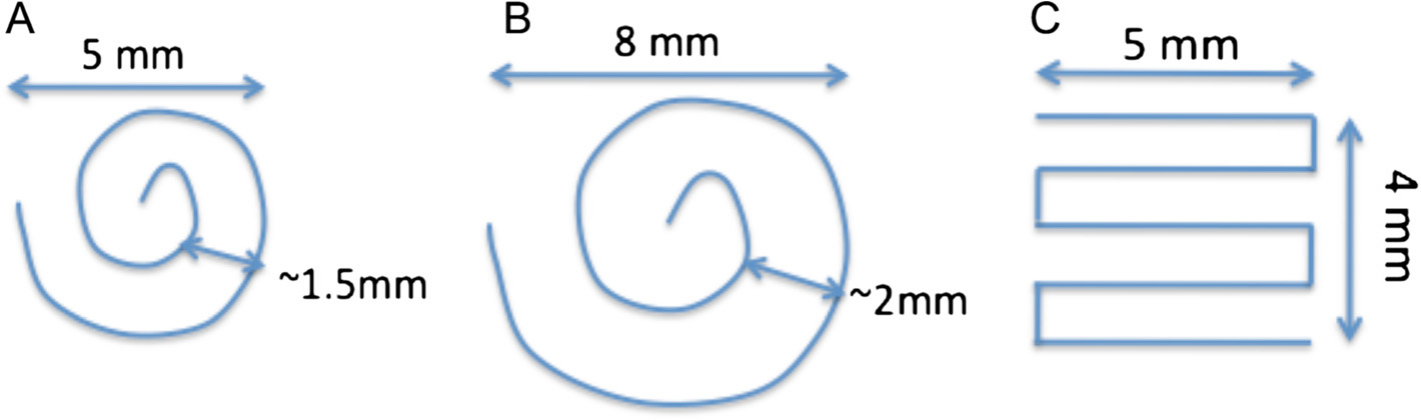
**Spiral and grid ultrasound beam patterns used in the study.** Transducer speed was 1 and 2.5 mm/s in the **(A)** 5-mm- and **(B)** 8-mm-diameter spiral patterns, respectively, and 0.1 mm/s in the **(C)** grid pattern.

The tumor was localized with high-resolution sagittal and axial images. Temperatures were monitored in a single coronal slice placed at the focal plane of the transducer. Three beam path patterns, two spiral patterns and one grid pattern, were used in these experiments (Figure 3). Transducer speed was 1 and 2.5 mm/s in the 5-mm- and 8-mm-diameter spiral patterns, respectively, and 0.1 mm/s in the grid pattern.

### Monitoring treatment outcome

Time lines of tumor growth or regression were documented using three complimentary monitoring techniques: tumor size measurements with a caliper, tumor fluorescence imaging, and photography. All three techniques produced similar results. Fluorescence imaging allowed the monitoring of both tumor growth/regression and cell death in the tumor tissue (see below).

Tumor volume was calculated using the following equation:

(1) $$V=\left(L\times W^{2}\right)/2$$

where *L* is the tumor length and *W* is the tumor width.

The end point corresponded to the tumor, reaching about 2 cm in diameter; the time to reach this point was taken as a life span.

### Animal groups

The treatment groups and number of animals used in the study are listed in Table 1. Experimental parameters included PTX-loaded nanodroplets vs. empty nanodroplets, applied MRgFUS pressure, and time of ultrasound application.

**Table 1:** Treatment parameters for all animals used in the study

***N***	**Treatment group**	**MRgHIFU parameters**				**Time between injection and MRgFUS (h)**	**Life span (weeks)**^**a**^
		**Trajectory**	**Acoustic pressure (MPa)**	**Duty cycle (%), pulse length**	**Total sonication time (s)**		
7	Negative control (no injection, no MRgFUS)	N/A	N/A	N/A	N/A	N/A	3.5 ± 0.5
7	PTX-nanodroplets + No MRgFUS						7.0 ± 0.8
1	No injection + MRgFUS	Spiral	3.4	100	240		4.8 ± 2.3
1			4.2		60		
4		Grid	3.4		325 ± 29		
4			4.2		300		
3	Empty nanodroplets + No MRgFUS	N/A	N/A	N/A	N/A	6 to 8	3.5 ± 0.5
1	Empty nanodroplets + MRgFUS	Spiral	3.4	100	60	6 to 8	3.5 ± 2.1^b^
2			4.2		60		
1			4.8		240		
4		Grid	3.4		313 ± 25		
4	PTX-nanodroplets + MRgFUS	Grid	3.4	100	300	1 to 2	7.0 ± 1.0
1			2.4				
4		Spiral	3.4	100	153 ± 54	6 to 8	10.3 ± 1.6^c^
4			2.4	100	130 ± 50		
1			4.2	100	60		
1			4.8	100	60		
5		Grid	3.4	100	300		
2			4.8	100	300		
2			4.2	100	300		
1		Grid	3.4	50 (50 ms)	300		6.0 ± 1.4
1		Grid	4.2	50 (50 ms)	300		
1		Spiral	3.4	50 (100 ms)	300		
1		Spiral	4.8	50 (50 ms)	300		

^a^Mean values plus/minus standard deviations are presented; ^b^Mice that died within several days after the treatment (observed for the MRgFUS pressure of or higher than 4.2 MPa) were excluded from the life span calculation; ^c^Survivors (*N* = 2 for the grid trajectory) were excluded from the life span calculation.

### Statistical treatment

The statistical significance of the differences between the pairs of groups were calculated using the two-tailed, two-sample equal variance *t* test; the differences were considered statistically significant for *p* < 0.05.

## Results

### Effect of the combined treatment with PTX-loaded nanodroplets and MRgFUS on tumor growth/regression and mouse life span

**Figure 4:**
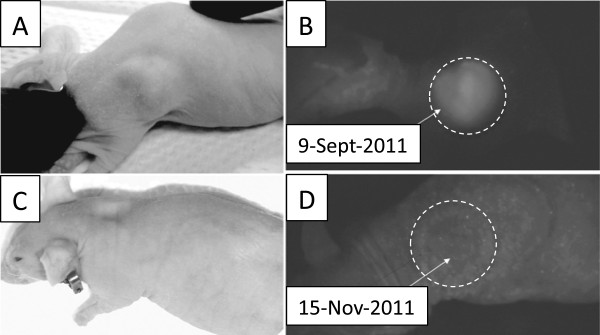
**Mouse photographs and whole-body fluorescence images before and after combined PTX-loaded nanodroplet and MRgFUS treatment.** Photographs **(A**, **C)** and whole-body fluorescence images **(B**, **D)** of a mouse before **(A, B)** and after **(C, D)** combined treatment with PTX-loaded nanodroplets and MRgFUS. The dashed circles in **(B,D)** indicate the tumor location. Treatment parameters: MRgFUS was applied 8 h after drug injection; spiral beam pattern (5-mm diameter) shown in Figure 3A; FUS at 3.1 MPa; sonication time of 3 min. The tumor did not recur during a 5-month observation. The former location of the tumor is still slightly visible in **D**, indicated by the dashed white circle.

Two different scenarios of the tumor response to treatment were observed: the first involved a complete tumor resolution without recurrence. This was observed in four mice after a single treatment with PTX-loaded nanodroplets and CW MRgFUS at an acoustic peak pressure of 2.4 or 3.4 MPa for either a spiral or grid beam trajectory. An example of a complete tumor resolution with both photograph and fluorescence images is presented in Figure 4.

**Figure 5:**
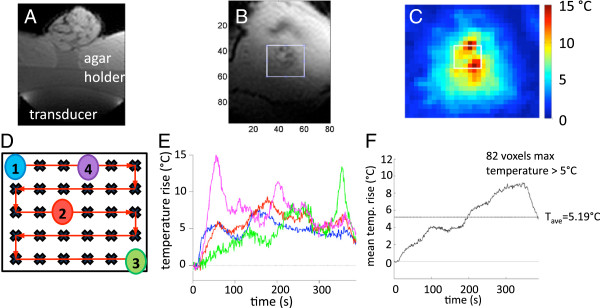
**Images of mouse, maximum temperature projection in time, MRgFUS trajectory, temperature response for voxels, and time curve. (A)** Axial image of a mouse on the MRgHIFU device. The transducer and agar holder are shown. **(B)** Coronal image through the tumor with region of interest defined. **(C)** Maximum temperature projection in time within region of interest. White rectangle indicates the grid trajectory boundaries. **(D)** MRgFUS trajectory with plotted voxel locations indicated. One-millimeter spacing between all points. **(E)** Temperature response as a function of time for voxels indicated in **(D)**. **(F)** Time curve of the mean temperature rise in the treatment plane for all voxels >5°C. Total MRgFUS time was 350 s at a pressure of 3.4 MPa.

The spiral steering patterns were executed with a much faster velocity than that of the grid pattern. This faster velocity in conjunction with the short MR acquisition time (approximately 1 s) led to unreliable temperature measurements from phase changes throughout the image caused by transducer motion. Grid steering patterns provided more robust temperature imaging during the treatment. An example of the temperature rise obtained with a grid steering pattern is presented in Figure 5. The tumor was treated with PTX-loaded nanodroplets and CW MRgFUS delivered for 350 s at 3.4 MPa. Panel (A) shows the axial image of a mouse on the MRgHIFU device. The transducer and agar holder are also shown. Panel (B) shows a coronal image through the tumor and the region of interest for the maximum temperature projection is shown in panel (C), in which the trajectory boundaries are indicated by the overlayed rectangle. The trajectory path is shown in panel (D) with voxel locations for the temperature response as a function of time plotted in panel (E). The mean temperature change (including all voxels that achieved a temperature rise of >5°C) in the treatment region is presented in panel (F).

The initial tumor was small (*V*_0_ = 164 mm^3^); after the treatment, the tumor regressed quickly and there was no tumor visible to eye or by RFP imaging. However, tumor regrowth started 6 weeks after the treatment. The recurrent tumor responded to a second treatment (nine injections of PTX-loaded nanodroplets without MRgFUS, twice a week for 4.5 weeks) indicating that tumor cells did not develop drug resistance. Histological examination of a tumor control showed the presence of mitotic cells and a pronounced stroma. In a recurred tumor in a mouse that received the combined treatment of PTX-nanodroplets and MRgFUS, no evidence of stroma and substantial necrosis in the residual tumor areas was observed. The presence of significant hemosiderin depositions in a treated tumor is a sign of the infarction of the initially treated tumor that appeared completely resolved and replaced with the scar tissue. For a different mouse with a larger initial tumor (*V*_0_ = 264 mm^3^) treated with the same protocol, complete tumor resolution took a longer time (8 weeks). The tumor did not recur during a 5-month observation.

The cases of complete resolution of pancreatic cancer occurred after a single pancreatic tumor treatment with PTX-loaded nanodroplets and MRgFUS. This was observed in four of twenty-eight mice treated with PTX-loaded nanodroplets with various MRgFUS parameters. Survivors were observed at ultrasound acoustic pressures of 2.4 (*N* = 1) or 3.4 MPa (*N* = 3) and did not depend on the beam steering pattern (i.e., spiral or grid).

**Figure 6:**
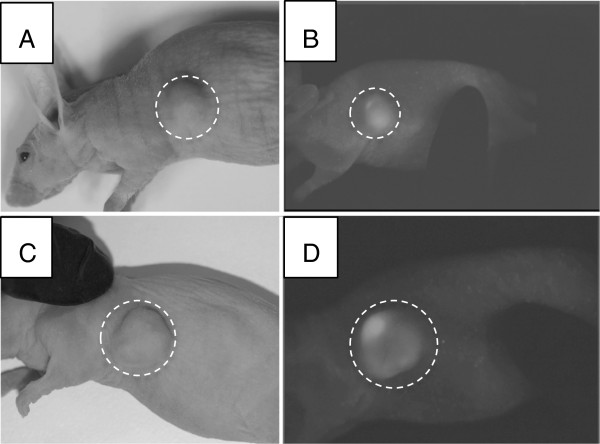
**Mouse photographs and whole-body fluorescence images before and after combined PTX-loaded nanodroplet and MRgFUS treatment.** Mouse photographs **(A**, **C)** and whole-body fluorescence images **(B**, **D)** taken before **(A, B)** and 3 weeks after treatment **(C,D)** with PTX-loaded nanodroplets and MRgFUS therapy; ultrasound was applied 8 h after drug injection at 2.4 MPa with a grid trajectory. The tumor location is indicated by a dashed circle in all images.

**Figure 7:**
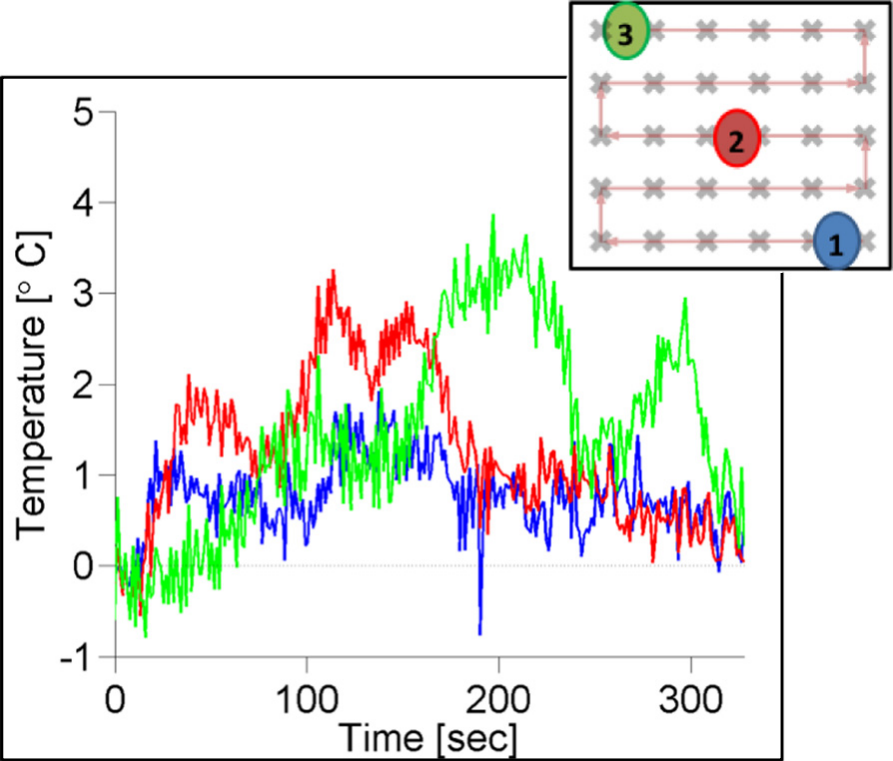
**Temperature response for the mouse shown in Figure **6**.** The temperature rise for three individual voxels indicated in the treatment path is shown.

A different tumor response scenario to the combined PTX-loaded nanodroplet and MRgFUS treatment is illustrated in Figure 6 for a mouse treated with the grid pattern at 2.4 MPa. The upper panel shows a mouse photograph and fluorescent image taken 1 h before the treatment; the lower photograph and image were taken 3 weeks later. This image suggests that tumor cells were killed in the MRgFUS-treated area but remained viable around the treated volume causing a gradual tumor growth around the treated area. The growth rate of this tumor was close to that of tumors not treated with MRgFUS. Temperature data for the animal shown in Figure 6 is displayed in Figure 7; a very marginal temperature response to MRgFUS therapy was observed.

**Figure 8:**
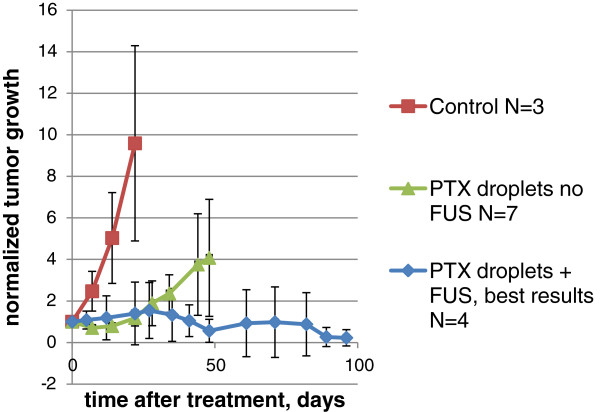
**Tumor growth curves.** Control (*N* = 3, squares); Tumors treated with PTX-loaded nanodroplets without ultrasound (*N* = 7, triangles); Best results for tumors treated with PTX-loaded nanodroplets and MRgFUS (*N* = 4, diamonds).

It is important to underline that the therapeutic effect of PTX-loaded nanodroplets was also observed without the MRgFUS treatment (Figure 8); however, complete tumor resolution has never been achieved after a single nanodroplet injection without MRgFUS, and the average mouse life span after a single treatment with PTX-loaded nanodroplets without ultrasound (7 ± 0.8 weeks, *N* = 7) was shorter than that of mice treated with MRgFUS (10.3 ± 1.6 weeks, *N* = 19, grid + spiral trajectory, survivors were excluded from the calculation, Table 1); the difference between these two groups was statistically significant (*p* < 0.05).

### The role of drug in the MRgFUS-mediated tumor treatment: comparison of the effects of empty and drug-loaded nanodroplets

Dramatic differences were observed in the tumor responses to MRgFUS treatment with and without drug. The MRgFUS tumor treatment without any injection did not affect tumor growth or mouse life span; any differences with control were not statistically significant.

Injections of empty (i.e., not PTX-loaded) nanodroplets without MRgFUS application or with MRgFUS pressure levels below 4.2 MPa did not exert any effect on the tumor growth rates or average mouse life span. Six mice were treated with empty nanodroplets of various MRgFUS pressure levels from 2.4 to 4.2 MPa; their average life span was 3.5 weeks, similar to the negative control; however, all mice treated with a pressure of 4.2 MPa died within 1 to 3 weeks after the treatment. In two cases, tumor growth was noticeably accelerated (data not shown). In contrast, the average life span of mice treated with PTX-loaded nanodroplets and MRgFUS was threefold longer (10.3 weeks). These data indicate that for the combined PTX-loaded nanodroplets/MRgFUS treatment, the main therapeutic effect was caused by the drug and not by the ultrasound. Still, as follows from Figure 8 and Table 1, MRgFUS did significantly enhance the action of the drug for certain combinations of ultrasound parameters.

### Effect of the ultrasound pressure

**Figure 9:**
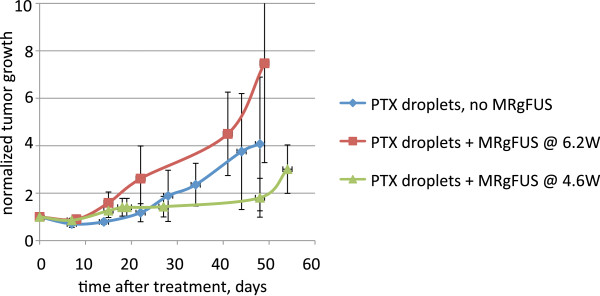
**Effect of ultrasound pressure on the tumor growth curves in the presence of PTX-loaded nanodroplets.** No MRgFUS (*N* = 7, diamonds); MRgFUS at 4.2 MPa (*N* = 3, triangles); MRgFUS at 4.8 MPa (*N* = 2, squares).

For mice treated with PTX-loaded nanodroplets and MRgFUS, increasing ultrasound pressure above 4.2 MPa exerted a detrimental effect on the tumor growth and animal survival (Figure 9); moreover, at a pressure level of 4.2 MPa and especially at 4.8 MPa, grid-shaped skin burns that required special treatment were observed. The burns were resolved within 2 to 3 weeks.

### Effect of the time of ultrasound application

Experiments were performed with ultrasound application either 2 (*N* = 5) or 6 to 8 h after the injection (*N* = 23). In one experiment, ultrasound was applied 10 min before the injection of PTX-loaded nanodroplets. No effect of MRgFUS was observed when ultrasound was applied either before or 2 h after the nanodroplet injection; tumor growth rates and average life span did not differ from those observed for PTX-loaded nanodroplets without MRgFUS.

### Effect of pulsed ultrasound

The experiments for pulsed and CW parameters were performed in parallel. The average life span of mice treated with pulsed ultrasound with various FUS parameters (6 ± 1.4 weeks, *N* = 4) was significantly lower than that of mice treated with CW ultrasound (10.3 ± 1.6 weeks, *N* = 19).

### Effect of the initial tumor size

The effect of the therapy depended strongly on the initial tumor size at the start of treatment. When the initial tumor size exceeded 1,000 mm^3^, the combined treatment by PTX-loaded nanodroplets and MRgFUS could not completely stop tumor growth; after the initial decrease of the tumor size, tumor growth resumed in 3 to 4 weeks. The average life span of animals with large initial tumors was increased by the treatment (roughly from 3 weeks for controls to 6 to 8 weeks for treated animals), but all tumors continued to grow despite the treatment. Increasing the MRgFUS-treated volume by the treatment of the two tumor planes rather than one plane did not exert any positive effect on the life span of animals with large initial tumors. Moreover, tumor growth was accelerated after the two-plane treatment, presumably due to the increased heating (see Discussion).

### Safety issues and collateral damage

**Figure 10:**
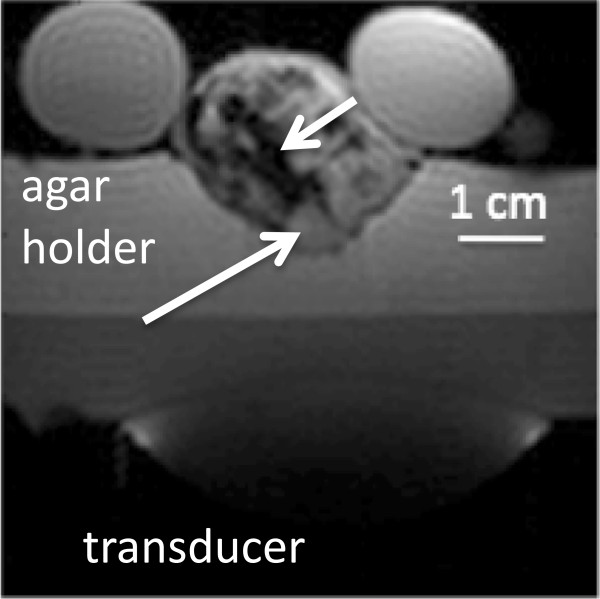
**MRI showing gas-filled intestines in the ultrasound far-field.** This image is representative for seven mice that died within several days after MRgFUS treatment. The transducer and agar holder are labeled. The long and short arrows identify the tumor and intestines, respectively.

Seven of the total of fifty-one animals (14%) treated with MRgFUS died *within several days* of the MRgFUS treatment. Four of seven animals died after the treatment with empty nanodroplets and MRgFUS at pressure levels of 4.2 or 4.8 MPa; two animals died after the treatment with the same MRgFUS parameters without any injection. One animal died two days after the combined treatment with PTX-loaded nanodroplets and MRgFUS at 4.2 MPa. No animal deaths resulted from the nanodroplet treatment without MRgFUS indicating that animal deaths were related to the MRgFUS treatment. Presence of empty nanodroplets during MRgFUS treatment appeared to increase the death rate. Although the exact mechanism that led to the animal's death is unknown, it is suspected that it may be due to peritonitis. A post-treatment analysis of MR images of coronal slices of MRgFUS treated animals suggested that the collateral damage occurred when gas-filled intestines were located in the far field of the ultrasound beam (Figure 10).

## Discussion

Ultrasound may exert both positive and negative effects on biological tissue. Positive effects may be related to increased drug carrier and drug extravasation, drug release from carrier, and drug internalization by tumor cells. On the other hand, vasodilation or vasoconstriction in response to ultrasound and cavitating microbubbles may result in the cellular response of surrounding tissues such as inflammation, edema, hemorrhage, which could be negative. Tissue response to ultrasound-induced heating is multifaceted as well. In the absence of the drug, the response to sub-ablative heating may be negative due to enhanced perfusion that promotes tumor growth. In contrast, in the presence of the drug, enhanced perfusion is a positive factor that promotes drug delivery to tumor tissue; in addition, hyperthermia increases vascular wall and plasma membrane permeability thus increasing drug internalization by tumor cells. The ultimate biological response to ultrasound presumably depends on the prevalence of positive or negative factors which is expected to depend on the treatment protocol as well as biological factors.

In our experiments, MRgFUS was applied at sub-ablative pressure levels and only a fraction of the tumor was treated. For tumor therapy, this situation is unfavorable. Ultrasound treatment caused hyperthermia (Figure 5) but did not induce coagulative necrosis of tumor tissue as manifested by the preservation of the tumor cell fluorescence after the MRgFUS treatment without the drug. At ultrasound pressure levels used in this study, MRgFUS treatment without any injection did not exert statistically significant effects on the tumor growth or animal life span. In the presence of empty nanodroplets, the effect of MRgFUS appeared to be negative, especially at higher pressure levels (increased tumor growth, early animal death), which could be related to the droplet-to-bubble transition and bubble cavitation that induced tissue inflammation. In the presence of the drug, these negative effects of the ultrasound treatment were suppressed. Moreover, the MRgFUS treatment enhanced the action of the drug (Figure 8 and Table 1). This may be associated with the enhanced tumor perfusion and cell permeability caused by mild hyperthermia, which in the presence of the drug are positive factors that increase drug supply to tumor cells.

However, increasing ultrasound pressure above a certain threshold (4.2 MPa) decreased the positive effect of the combined PTX-loaded nanodroplet/MRgFUS treatment (Figure 9). This shows that positive effects of the ultrasound treatment in the presence of nanodroplets compete with negative effects in this animal model; adverse effects causing tissue inflammation are enhanced at higher ultrasound pressure levels. Even in the presence of the drug, one animal died within a couple of days after the MRgFUS treatment at 4.2 MPa. However, the death rate caused by the MRgFUS treatment was much higher with empty droplets.

It is not immediately clear if the chemotherapeutic drug may suppress the inflammation. One hypothesis was suggested by Dr. Klibanov who proposed that the nanodroplet-encapsulated PTX could kill harmful macrophages that cause tissue inflammation (personal communication). This supposition remains to be explored.

As suggested by the post-treatment analysis of MR images, ultrasound-induced collateral damage could result from ultrasound reflection from the air-filled structures (Figure 10); possible intra-intestinal gas bubble cavitation in the ultrasound far field could be a peritoneum-damaging factor that results in the animal death.

In the presented experiments, no correlation was observed between the mean temperature in the treated plane and the treatment outcome. More data are needed to draw a conclusion on the relative roles of thermal and mechanical modes of ultrasound action in the combined treatment with PTX-loaded nanodroplets and MRgFUS.

The effect of the pulsed MRgFUS treatment with PTX-loaded nanodroplets was clearly smaller than that of CW MRgFUS treatment, which may be related to significantly lower temperature increase and/or decreased extravasation [50].

The time of the MRgFUS application proved to be an important factor. The positive effect of the MRgFUS treatment in the presence of PTX-loaded nanodroplets was observed when MRgFUS therapy was performed 6 to 8 h after the injection of drug-loaded nanodroplets but was aborted when MRgFUS was applied before or shortly after the drug injection. This underlines the role of the nanodroplet accumulation in the tumor volume for the success of the treatment. The data suggest that at the conditions studied, MRgFUS treatment did not noticeably enhance nanodroplet extravasation into the tumor tissue. The opposite was observed in a recent study where excised carotid arteries were inserted in PBS [50]. It appears reasonable to suggest that the ultrasound susceptibility of blood vessels inserted in solid matrices could differ from that of vessels inserted in liquid. It might well be that enhanced extravasation *in vivo* requires higher ultrasound pressures than those used in this work. At this time, we do not have enough data to further explore this issue. The data presented also show that nanodroplet accumulation in the tumor is desirable before the ultrasound application. The tumor accumulation of nanodroplets may take hours [21,22]. The presence of nanodroplets in blood vessels of the normal tissue in the path of the beam may scatter ultrasound; it may also cause collateral damage induced by the microbubble cavitation in the ultrasound far field.

Since increasing the sonicated volume did not result in a better treatment outcome for large tumors, the inability to completely suppress tumor growth was most probably related to the insufficient concentration of drug in the tumor rather than the small sonicated tumor fraction.

The data presented above suggest that there exists an optimal range of the time of ultrasound application and ultrasound pressure levels below which the treatment does not exert any effect and above which the effect of the treatment is decreased. It should be emphasized that positive treatment effects and even complete tumor resolution were achieved with only a single treatment of just a fraction of the pancreatic tumor. This shows the potential of using the combined tumor treatment with PTX-loaded nanodroplets and MRgFUS for the therapy of the pancreatic cancer, one of the most lethal cancer types.

## Competing interests

The authors declare that they have no competing interests.

## Authors' contributions

NR conceived the study, designed and coordinated the project, participated in all experiments, processed the data, and drafted the manuscript. AP designed and ran MRgFUS experiments, participated in data processing, and edited the manuscript. CD participated in MRgFUS experiments, processed MR thermometry data, and participated in editing the manuscript. JS inoculated RFP-transfected pancreatic tumors and participated in MRgFUS experiments. CS participated in discussions of data. RG participated in all experiments, measured tumor sizes, took RFP images, and participated in data processing. All authors read and approved the final manuscript.
